# Functionality Enhancement of Industrialized Optical Fiber Sensors and System Developed for Full-Scale Pavement Monitoring

**DOI:** 10.3390/s140508829

**Published:** 2014-05-19

**Authors:** Huaping Wang, Wanqiu Liu, Jianping He, Xiaoying Xing, Dandan Cao, Xipeng Gao, Xiaowei Hao, Hongwei Cheng, Zhi Zhou

**Affiliations:** 1. School of Civil Engineering, Dalian University of Technology, Dalian 116024, China; E-Mails: wanghuaping1128@sina.cn (H.W.); hejianping_hit@163.com (J.H.); xipeng1004@126.com (X.G.); hao987129688@sina.com (X.H.); chenghwdy@126.com (H.C.); 2. Department of Transportation and Logistics, Dalian University of Technology, Dalian 116024, China; E-Mails: liuwanqiu@dlut.edu.cn (W.L.); 18940920128@163.com (X.X.); cdd1017@163.com (D.C.)

**Keywords:** pavements, functionality enhancement of industrialized optical fiber sensors, self-healing network system, full-scale monitoring

## Abstract

Pavements always play a predominant role in transportation. Health monitoring of pavements is becoming more and more significant, as frequently suffering from cracks, rutting, and slippage renders them prematurely out of service. Effective and reliable sensing elements are thus in high demand to make prognosis on the mechanical properties and occurrence of damage to pavements. Therefore, in this paper, various types of functionality enhancement of industrialized optical fiber sensors for pavement monitoring are developed, with the corresponding operational principles clarified in theory and the performance double checked by basic experiments. Furthermore, a self-healing optical fiber sensing network system is adopted to accomplish full-scale monitoring of pavements. The application of optical fiber sensors assembly and self-healing network system in pavement has been carried out to validate the feasibility. It has been proved that the research in this article provides a valuable method and meaningful guidance for the integrity monitoring of civil structures, especially pavements.

## 1. Introduction

A large number of structures built decades ago are in urgent need of strengthening, rehabilitation and replacement, and structural health monitoring (SHM) has emerged as a diagnostic tool to monitor *in-situ* structure behavior accurately and efficiently [1–4]. A SHM system comprised of various components, covering optical fiber (OF) sensors, is utilized to identify damages, assess performance, predict residual service life of structures and give real-time sound warnings, which has been recognized as an efficient approach for saving lives and reducing economic losses [5,6]. It is expected that SHM will take shape into an approach, which stands shoulder to shoulder with traditional methods, namely theory, experiments and numerical analysis, to extract the mechanical properties of structures.

Among these components, OF sensors and Fiber Bragg grating (FBG) sensors, for the advantages of long-term stability and durability, good geometrical shape-versatility, corrosion resistance, anti-electromagnetic interference, low cost and high precision detection, have found wide-spread application [7–11]. Research on OF sensors has extended into diverse technological fields, not only covering aerospace, ocean platforms, underground construction and transportation engineering, but also including the medical, chemical and telecommunication industries [12]. OF sensors have been designed to measure a wide variety of physical properties, such as chemical changes, strain, electric and magnetic fields, temperature, pressure, rotation, displacement (position), radiation, flow, liquid level, vibration, light intensity and color [12–14]. FBG sensors packaged with different materials have been manufactured and successfully utilized to detect humidity, temperature, strain, cracks and acceleration in aeronautics, energy, railway, nuclear environmental fields and so on [15–18]. A small part of the functions of the two types of sensors overlaps, but still there are distinguishing characteristics that OF sensors highlight the distributed inspection and FBG sensors majorly focus on high-precision local measurements [19–24].

Since pavements are constituted of asphalt/concrete mixtures and gravels with different particle sizes, and are thus considered heterogeneous structures, it is quite difficult to develop much accurate theory and numerical methods to depict these mixtures' non-uniformity [25–27], while the expense of batch tests is huge, and results obtained with them usually don't match well with experimental measurements [28]. SHM technology is a potentially feasible approach to capture real information, and it has received many researchers' approval [29–32]. The composition uncertainty, temperature sensitiveness and viscoelasticity characteristics of pavement materials make the pavement structural analysis very complex, compared with other civil structures, such as bridges and buildings. Evaluation of the performance of existing pavements is a priority issue, as it is very hard to devise an efficient method to determine realistic mechanical properties [33]. The layered elastic theory, ignoring the uneven, anisotropy and nonlinear stress-strain relationship of paving materials, just offers calculation results incongruent with the real state of pavements [34]. For this reason, there has been interest in improving all kinds of sensors so as to exhibit strain, stress and displacement with much higher precision, which would provide a reliable scientific basis for modification of the theory.

Scholars have contributed to the application of OF sensing technology in large-span pavements, and some foundational achievements have been obtained. Various packaging methods for OF and FBG sensors were investigated, including steel bar [35], fiber reinforced polymer (FRP) [36], steel sheet [37], bending device [38], pipe [39], polypropylene (PP) [40,41], glue [42,43], geotextile [44] and cables [45]. Optical fiber strain gauges with a retrofit technique measured strains in the upper and lower part of the asphalt layer [46]. An OF sensor based on Fabry-Perot (F-P) technology was introduced to detect strain of cold in-depth recycled utilizing foamed asphalt [47]. These sensors could realize their function to some extent, but employing steel pipe, FRP, and PP as cladding material, the coordinate deformation between protective layer and asphalt mixture couldn't be well resolved, which directly led to low precision. Selecting appropriate materials to match the modulus of asphalt mixtures and simultaneously guaranteeing the survival of sensors so far has been treated as a bottleneck issue. Consequently, novel improved sensors that could easily survive in pavement construction and possess commendable coordination deformation with the host material are in high demand, which would serve for high-precision detection and real mechanical-parameter acquirement of pavements.

Due to the imperfections and incompleteness of subsensors, full-scale monitoring of multi-layered pavements has seldom been mentioned [48], while pavements usually suffer from random damage, and local destruction without timely maintenance often results in failure of large areas. Therefore, assembling these subelements, particularly to establish a self-healing network system is a necessity [49], which would assist in accomplishing full scale deformation detection and eventually serve for pavement evaluation and inverse optimization design.

Given the analysis above, various types of functionality enhancement of industrialized optical fiber sensors developed for pavement monitoring are put forward in this article for the first time, and the corresponding operational principles are also demonstrated and the performance checked by basic experiments. A self-healing OF sensing network system is adopted and distributed sensors embedded in a single layer structure are implemented to support the feasibility studies. Moreover, a study of a three-layered asphalt pavement embedded with armoring pipe packaged FBG and OF sensors is conducted on site to check the influence of environmental temperature changes on asphalt pavement strain.

## 2. Functionality Enhancements of Industrialized Optical Fiber Sensors

In addition to satisfying the basic principles mentioned above, the design of sensors should also take the unique features of pavements into account. That is to say, sensors developed for pavement behavior monitoring must reckon with the mechanical properties of material, structure and damage mode simultaneously as follows:

(1) For the material, its composition, temperature influence and viscoelasticity should be considered;
(2) For the structure, as a multi-layered system with different media in each layer, the long span feature of the pavement makes it cross over different geological stratums, and the hierarchical and distributed distinction should be considered;
(3) For damage modes, the causes and appearances of different damage modes are influenced by the characteristics of the material and structure, as stated before, with cracks, rutting and subsidence being the most common types.

During the paving process, the protective layers of sensors also need to resist large compaction forces and high temperatures. Besides, the cost of sensors cannot be high due to the large-scale installation.

Therefore, the functionality enhancements considered in this paper for industrialized optical fiber sensors, including FBG sensors for high-precision local detection and OF sensors for distributed sensing using raw materials (fine aggregate mixture and asphalt mixture) and armoring wires have been developed. Details are discussed in the following sections.

“Standard Test Methods of Bitumen and Bituminous Mixtures for Highway Engineering” [50] and “Test Methods of Aggregate for Highway Engineering” [51] are the main references of the experiments carried out in this paper.

### 2.1. Experimental Equipment and Operational Principles

**Figure 1.**
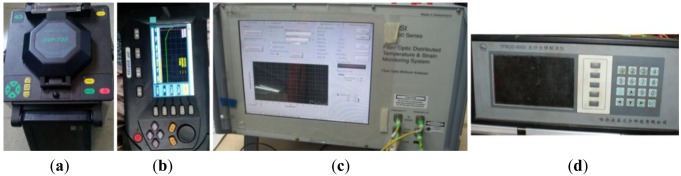
Equipment used in optical fiber sensing experiments. (**a**) Fusion splicer; (**b**) OTDR; (**c**) BOTDA; (**d**) FBG interrogator.

Equipment used in the experiments is mainly composed of welding and demodulation devices. In the fabrication process, a fusion splicer is adopted to connect the optical fiber and patch cords, aided by optical time domain reflectometry (OTDR) (Nanjing DVP optical & Electronical Tech. Co. Ltd., Nanjing, China) to detect any light discontinuities. Images are displayed in Figure 1a,b. During the sensing period, Brillion Optical Time Domain Analysis (BOTDA) (Micro Optics, Hackettstown, NJ, USA) is employed to interpretate the frequency shift signal of the optical fiber sensors, and a FBG intterogator (Harbin Teda Tech. CO. Ltd., Harbin, China) is used to to abstract the wavelength changes. These are shown in Figure 1c,d, respectively.

#### 2.1.1. Operational Principle of Brillion Optical Time Domain Analysis (BOTDA)

**Figure 2.**
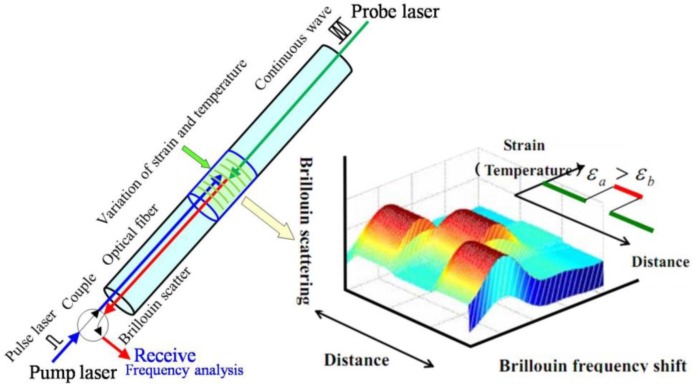
Operational principle of the optical fiber sensor and the Brillouin gain spectrum.

BOTDA is a technique based on simulated Brillouin scattering caused by acoustical phonons which results in a frequency shift [52,53], as displayed in Figure 2. Two laser sources, one a pump (pulse) laser source and the other a probe laser source, are introduced into optical fiber from two ends. When the frequency difference between the two lasers is equal to the Brillouin frequency shift, the back Brillouin scattering is simulated [53]. It has been found that the Brillouin shift of optical fiber is linearly related to applied strain and temperature. BOTDA is one of the demodulating systems used to obtain distributed strain or temperature measurements along the fiber by using the good linear relationship [54] between the Brillouin frequency shift and strain/temeprature expressed by the function:

(1) $$\nu_{B}(T,ɛ)=\nu_{B0}(T_{0},{ɛ}_{0})+C_{ɛ}ɛ+C_{T}\Delta T$$

where, *ν_B_*, *ν_B_*_0_, *C*_ε_ and *C_T_* indicate the Brillouin frequency shift, original Brillouin frequency shift, strain and temperature coefficients, respectively.

#### 2.1.2. Operational Principle of Fiber Bragg Grating (FBG)

**Figure 3.**
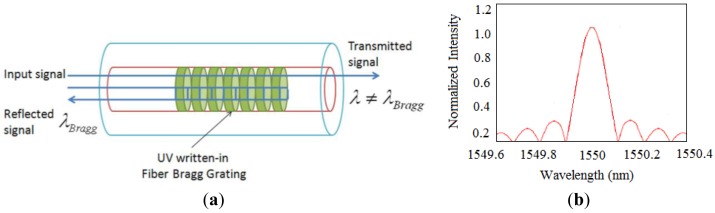
Operation principle of the FBG sensor and a typical spectrum. (**a**) The signal interrogation system of FBG; (**b**) Typical spectrum of FBG sensor.

FBG are made by laterally exposing the core of a single-mode fiber to a periodic pattern of intense ultraviolet light. The exposure produces a permanent increase in the refractive index of the fiber's core, creating a fixed index modulation called a grating according to the exposure pattern [36]. At each periodic refraction change, a small amount of light is reflected. All the reflected light signals combine coherently into one large reflection at a particular wavelength when the grating period is approximately half of the input light's wavelength [36]. Referred to as the Bragg condition, the wavelength at which this reflection occurs is called the Bragg wavelength. Light signals at wavelengths other than the Bragg wavelength, which are not phase matched, are essentially transparent [36], as shown in Figure 3a. Therefore, light propagates through the grating with negligible attenuation or signal variation. Only those wavelengths that satisfy the Bragg condition are affected and strongly back-reflected. Figure 3b shows the typical output reflected spectrum of FBG [36,55]. The central wavelength of the reflected component satisfies the Bragg condition:

(2) λ=2nΛ

where *n* is the index of refraction and Λ is the grating periodicity.

Due to the temperature and strain dependence of the parameters *n* and Λ, the wavelength of the reflected component will change as a function of temperature and strain. The general expression of the strain-temperature relationship for a FBG strain sensor can be described by [56]:

(3) $$\frac{\Delta \lambda }{\lambda }=(1-P_{ɛ})ɛ+(\alpha +\zeta )\Delta T$$

where λ, ξ, α, *P*_ε_ and *T* are the wavelength, thermal-optics coefficient, thermal expansion coefficient, optical elasticity coefficient and temperature, respectively.

### 2.2. Fine Aggregate-Asphalt Mixture Packaged Strain Sensors for High-Precision Monitoring of Pavements

**Figure 4.**
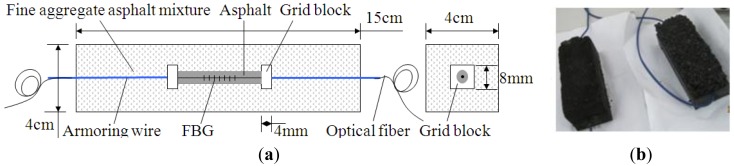
A sketch and actual photo of a fine aggregate-asphalt mixture packaged FBG sensor. (**a**) Layout of the FBG sensor; (**b**) Physical model.

It is observed that the elastic modulus of protective layer should match with that of host material, and then, raw material-encapsulated optical fiber sensors have been provided [57]. The first type of raw materials used is fine aggregate asphalt mixture. Fine aggregate-asphalt mixture packaged FBG sensor with width and length size suggested is listed as Figure 4a and physical model displayed in Figure 4b. Gradation of the fine aggregate asphalt mixture is shown in Table 1. As bare FBG is very weak and the surface of fiber is smooth, a thin pure-asphalt layer is added to protect FBG and establish good bonding with fine aggregate asphalt mixture. Strain of host material, ε*_m_*, passing through protective layer (viz. fine aggregate asphalt mixture), makes the distance between two grid blocks elongation, and then wavelength of FBG, λ, changes.

**Figure 5.**
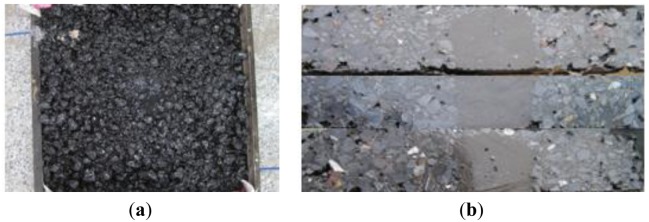
Cross-section of a wheel rutting test sample with the sensor embedded. (**a**) Wheel rutting test sample with optical fiber embedded; (**b**) Cross section of the test sample.

A wheel rutting test sample (300 mm × 300 mm × 50 mm, AC16) embedded with this sensor has been produced, and integrity of the fine aggregate-asphalt mixture packaged sensor, with the cross-section shown in Figure 5, has been confirmed after the rolling compaction process. It indicates that this sensor could survive the paving process, as the outside interfaces are tightly agglutinated with those of the asphalt mixture, which means the use of fine aggregate-mixture packaged FBG sensors for asphalt pavement monitoring is feasible.

**Figure 6.**
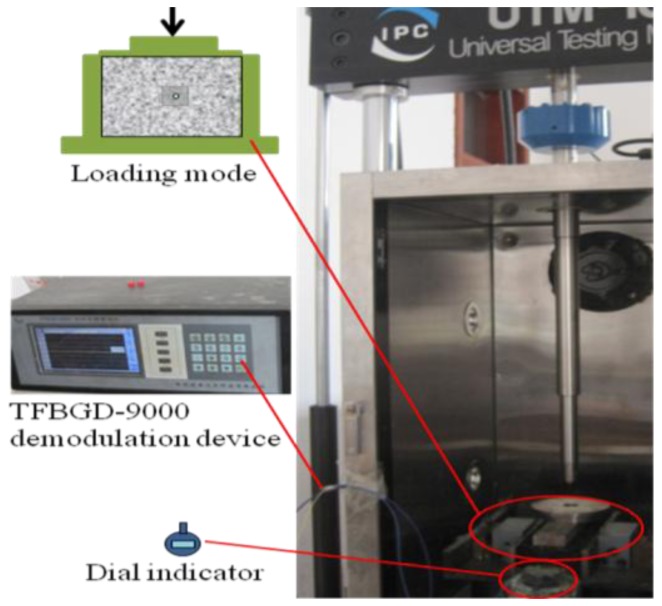
System loading device.

After making a cuboid with this embedded FBG sensor, and restraining side surfaces and imposing step loads by Universal Test Machnine (UTM), the resulting system is as displayed in Figure 6.

**Figure 7.**
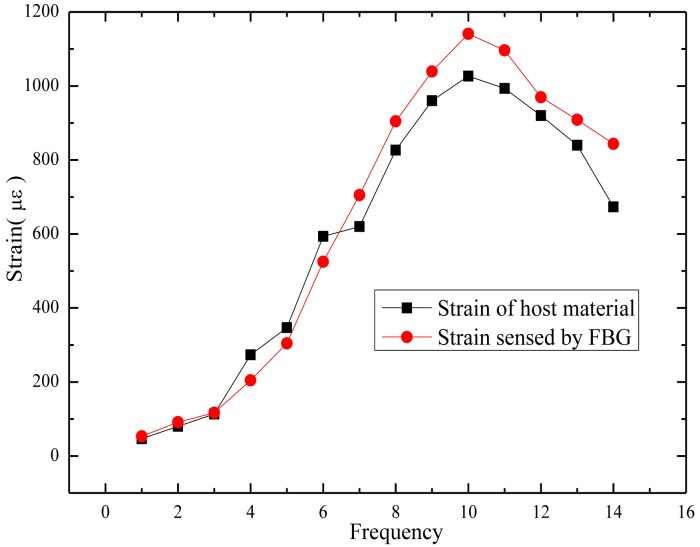
Strain detected by the FBG and the dial indicator.

A FBG interrogator has been employed for collecting strain data and a dial indicator introduced to abstract the average transverse displacement. Displacement increments measured by the dial indicator are tranformed into strains by the linear strain theory, and the transformed strains stand for the strain of host material, ε*_m_*, used for proofreading. The relationship between the strain detected by the FBG, ε*_f_* and the strain of host material, ε*_m_*, is illustrated in Figure 7. The results demonstrate that this sensor could provide reliable measurements. The tiny discrepancy is mostly composed by two parts. One part results from the strain loss consumed in the transfer path, which is usually called strain transfer error [58], and could be eliminated by introducing the modification equation [59]:

(4) $${ɛ}_{fa}(x)=\{1-\sinh(\lambda_{0}L)[\lambda_{0}L\cosh(\lambda_{0}L)]\}\cdot {ɛ}_{m}$$

where, λ_0_ is constant and *L* stands for the gauge length.

Another part comes from the average of the displacement increase detected by the dial indicator; it represents a strain along the axis of the cuboid that is equivalent at every point, while the real state is that the strain is larger in the center and smaller at the two ends, which contributes a lot to the discrepancy. As the relationship of transverse and vertical strain of asphalt mixture is uncertain and influenced by a lot of factors, such as compactness, it is thus difficult to get a high-precision strain value which approaches the real strain of asphalt mixtures. All these facts augment the error. A much higher-precision test could be obtained by adopting other devices to obtain the real strain of asphalt mixtures.

### 2.3. Asphalt-Mastic Packaged OF Strain Sensor for Distributed Monitoring of Asphalt Pavements

**Figure 8.**
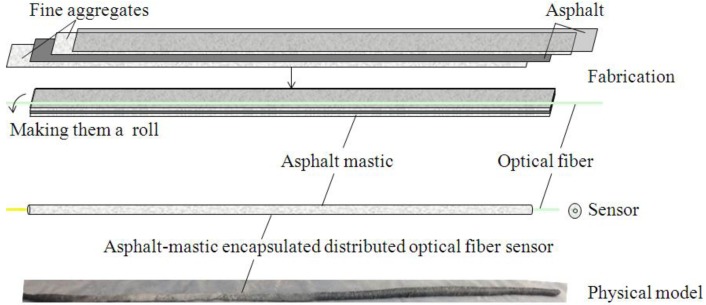
Manufacturing process of an asphalt-mastic encapsulated distributed OF sensor.

An asphalt-mastic packaged OF sensor, whose corresponding manufacturing operation and appearance are shown in Figure 8, has been designed for distributed monitoring of flexible pavement [60]. The asphalt mastic gradation is listed in Table 2.

**Figure 9.**
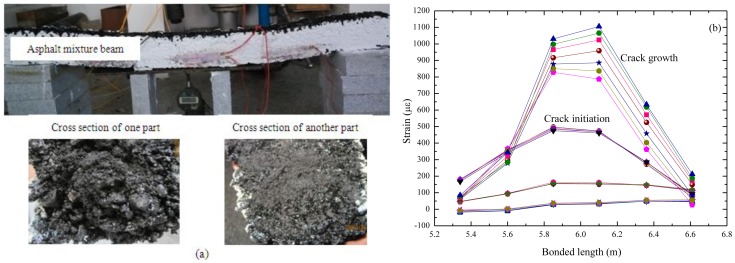
Asphalt mixture beam embedded with an optical fiber sensor. (**a**) Experimental set-up; (**b**) The corresponding data.

The strain of the host material, ε*_m_*, passes through the interfaces (viz. the interfaces between the asphalt mixture, asphalt mastic and optical fiber), and then, arriving at the optical fiber, it makes the fiber core elongate. 1.5 Meter-length beams with this sensor embedded have been created and support a uniform gravity load, with BOTDA being utilized for collecting the distributed strain data. The experimental set-up and cross-section of the asphalt mixture beam follows Figure 9a, and the corresponding data are shown in Figure 9b. In the first stage, no cracks occur on the beam and the strain line appears smooth, with a maximum value of less than 100 με. When a crack occurs at the center, a sudden growth of the strain line emerges. With the expansion of crack size, the corresponding strain data increases. The test results illustrate that this sensor is efficient for distributed monitoring of asphalt pavements, which means this flexible asphalt-mastic encapsulation technology is feasible for this use.

### 2.4. Armoring-Wire Encapsulated FBG Sensor with Spring for Large-Strain Monitoring of Pavements

**Figure 10.**
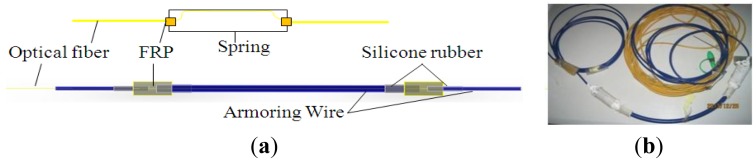
Sketch and actual photo of an armoring-wire encapsulated FBG sensor. (**a**) Structure of the FBG sensor; (**b**) Physical model.

Armoring wire has been selected as the encapsulation material, as it is able to bear strong compaction forces, but bends freely. Two grid blocks made of FRP are fixed at both sides of FBG. Silicone rubber gaskets are introduced to connect the FRP blocks and armoring. A spring is added to the sensing element to decrease the sensitivity and realize an enlarged test range. The structure and a physical model of the armoring- wire encapsulated FBG sensor with a spring in series are shown in Figure 10.

**Figure 11.**
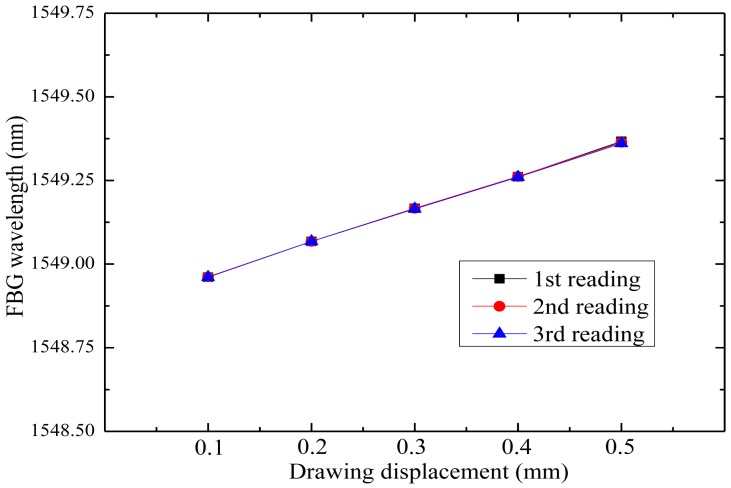
Tension test data of a FBG connected in series with a spring.

The influence of the spring on the strain detected by the FBG has been tested in the laboratory, and good linearity is observed, as shown in Figure 11. The results show that the FBG receives half of the strain detected by the sensing element, which means the sensitivity is a half compared with a bare FBG.

**Figure 12.**
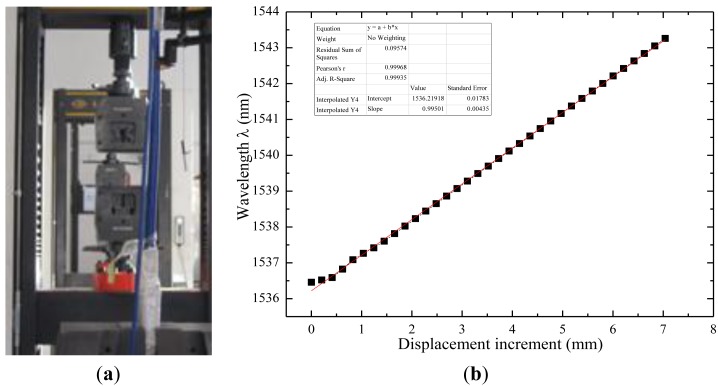
Tension test and strain data of the armoring-wire encapsulated FBG sensor. (**a**) Tension test; (**b**) The fitted data.

The performance of the sensor has also been tested in a material testing machine with force control, as shown in Figure 12a. Supplemental tension experiments by displacement control have been accomplished to detect the behavior of this FBG sensor. The interrogator has been used to collect strain data when continuous force and step displacement increments are applied on the FBG. Eight cycles with gradual displacement increases have been executed to check the parameters of this sensor, and the fitted data is shown in Figure 12b. The Pearson's coefficient *r* is higher than 0.99, which demonstrates good linearity of this FBG sensor. Sensitivity parameter is stable at 0.8 pm/με, while the common strain-sensing coefficient of FBG is 1.2 pm/με. For a 20 cm-length gauge FBG sensor, the tension test illustrates that the measurement range of this armoring-wire packaged FBG sensor is up to 35,200 με. Results indicate that this sensor behaviors well under cyclic loads and could bear a much larger strain in a normal state.

### 2.5. Armoring-Wire Encapsulated Quasi-Distributed OF Sensors for Large-Span Monitoring of Pavements

**Figure 13.**
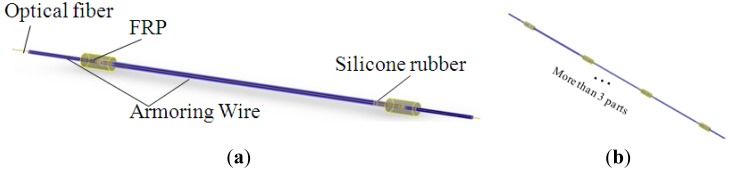
Sketch of a quasi-distributed OF sensor. (**a**) Structure of single sensing part; (**b**) Assembled quasi-distributed sensor.

Considering the advantages of the armoring wire setup mentioned above, armoring-wire encapsulated quasi-distributed OF sensors have been developed. The outstanding feature of this sensor is the division of the distributed optical fiber into continuous discrete sensing parts, as shown in Figure 13b. As armoring-wire encapsulated FBG sensor has been proved to function as intended, this quasi-distributed OF sensor could be quite likely to be feasible in engineering applications.

**Figure 14.**
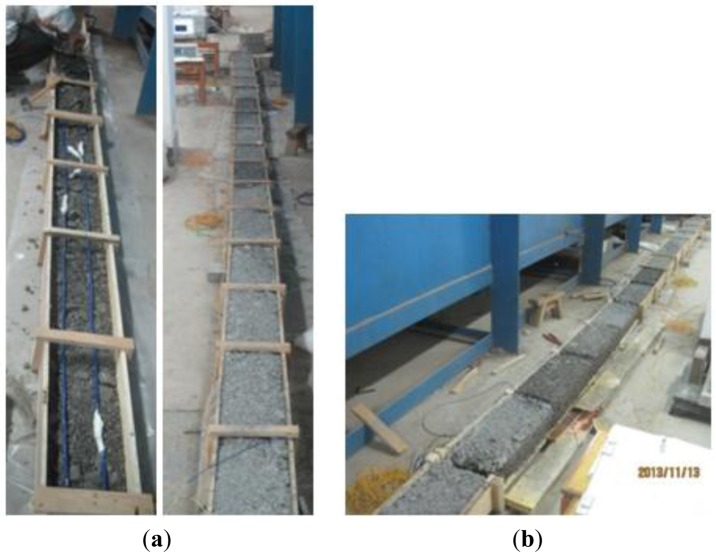
Cement stabilized layer embedded with quasi-distributed OF sensors. (**a**) Sensor layout and structure; (**b**) Cement structure with large cracks.

An 8-meter stabilized cement layer embedded with this quasi-distributed OF sensor has been prepared in the laboratory, and cracks have been added to examine its performance, as displayed in Figure 14.

**Figure 15.**
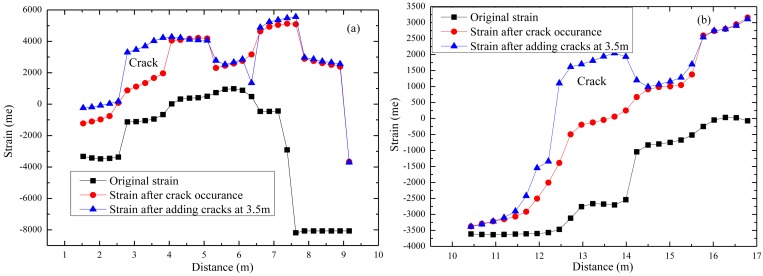
Strain detected by the proposed sensor along the span. (**a**) Data sensed by 1.2-meter sensing part; (**b**) Data sensed by 1.5-meter sensing part.

BOTDA has been employed to collect strain data. Part of the typical data is shown in Figure 15. Results shows that local cracks just cause strain changes of the corresponding gauge, which means this armoring-wire encapsulated quasi-distributed OF sensor could be used to identify local damage. Compared with many other FBG sensors, this sensor costs very little. Compared with the common distributed OF sensor, the mode of action of this sensor eliminates the influence of surrounding OF on the sensing part located in the middle, which indirectly improves the measuring precision. This proposed quasi-distributed optical fiber sensor simultaneously realizes much higher precision and multi-scale distributed monitoring.

## 3. Self-Healing OF Sensing Network System Used for Full-Scale Monitoring of Pavements

Based on the definition of survivability relative to optical communication, the self-healing structural health monitoring network is redefined as follows: “SHM sensing networks still maintain connectivity and the ability of local and overall safety evaluation after the network undergoes various fault (e.g., physical faults and software faults), such as the failure of local sensors, the damage of the optical fiber and the signal mutual interference, even a disastrous fault such as an earthquake, explosion or fire.” Following this new definition of survivability of structural health-monitoring networks, design principles of the self-healing OF sensing network are as follows: (a) Protection: the sensors and the sensing line must be protected against man-made destruction and from its own failure through structural damage; (b) Rapid diagnosis: the faults should be detected and located in time, at the moment when faults in the sensing network occur; (c) Robustness: the sensing network should have strong redundancy that is sufficient to maintain connectivity and measuring continuity based on some self-healing algorithms, especially in structurally vulnerable areas; (d) Artificial repair: to guarantee the measurement continuity of key points, new sensors and optical fibers can be repaired at the position of node failure in the network; (e) Capability for network reconstruction: the repaired nodes must be compatible with the network before conducting repairs to hardware (e.g., equipment connection) and software (e.g., SHM safety assessment).

**Figure 16.**
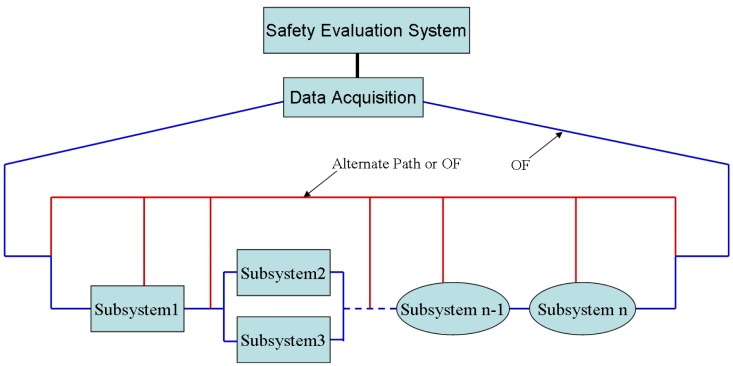
A self-healing sensing network.

Due to the large scale of the dimensions and the geometrical complexity of a typical civil structure, a large number of sensors are required to measure various structural and environmental parameters, which make the SHM system complex. One SHM network can be divided into numerous subsystems, each of which has a self-healing functionality. The subsystems are connected to one another by an armored OF, and the OF is kept free even when the structure is under large deformation. Furthermore, one alternate OF or laser path is needed to link the good subsystems to the data acquisition system when any sub- system or part of the armored OF fails, as shown in Figure 16. Each subsystem can be a spider-OF sensing network (OFSN) or OF-based hybrid system. The whole SHM system can be a large spider-OFSN, regarding each subsystem as a local spider-OFSN [61].

**Figure 17.**
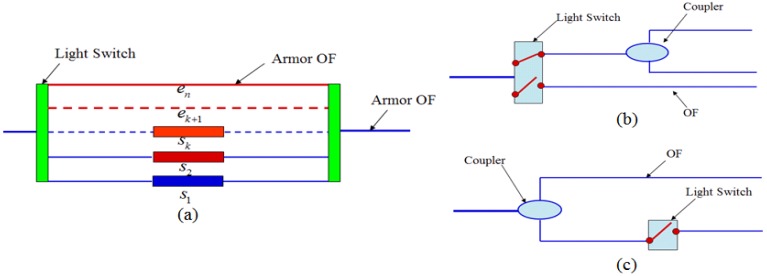
Self-healing sensor nodes. (**a**) Type-I; (**b**) Type-II; (**c**) Type-III.

Furthermore, to enable the network to find the new access automatically, three types of self-repair nodes are designed, as shown in Figure 17. Among these nodes, the self-repairing node Type-I includes two smart light switches, sensors (s1, …, sk), and an armored OF (ek + 1, …, en). The number of sensors and armored OF is determined based on the degree of connectivity of the local position. All sensors in the self-repair nodes are intercorrelated. The sensor si begins to work after the sensor si-1 fails, and the initial value of si is equal to the last value measured by the sensor si-1. The armored OF (ek + 1, …, en) maintains the network connection after all sensors fail. The smart light switch can automatically choose a light path from the bottom port to the top port if the sensing path is found broken. The self-healing sensor nodes Type-II and Type-III consist of one light switch and one coupler. As shown in Figure 17, when the OF path is broken, the light switch begins to form a new OF path. A complex self-healing network can thus be constructed by combining a large number of self-healing sensor nodes.

**Figure 18.**
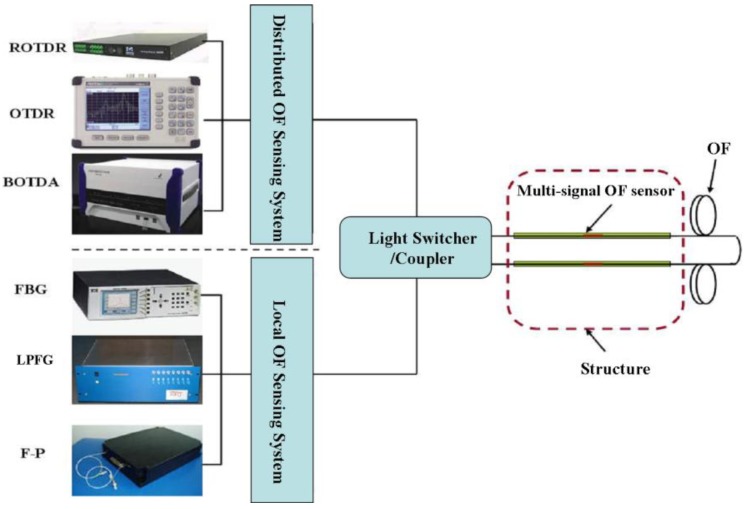
Schematic configuration of an OF hybrid system.

Figure 18 shows the schematic configuration of a local and distributed OF hybrid system, which consists of local OF sensing systems (FBG, F-P and long period fiber Bragg (LPFG )) and distributed OF sensing systems (BOTDA, Romain optical time domain reflector (ROTDR) and OTDR), light switcher or coupler, multi-signal OF sensors (e.g., BOTDA-FBG sensor) [61]. As the local sensors are installed on the stress hot area for high-precision measurement and the distributed sensors are installed to obtain global information of a structure, the hybrid system can provide multi-signal in one OF sensor.

**Figure 19.**
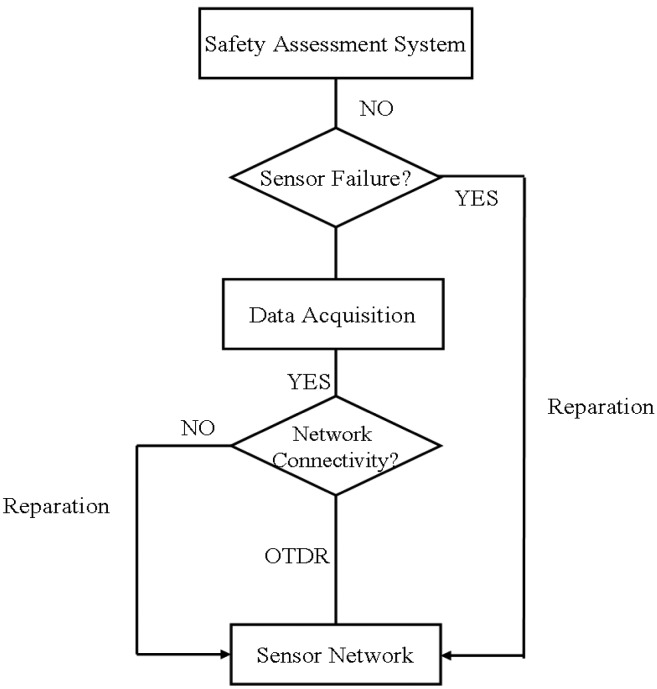
Repair strategy of self-healing network.

When damage occurs in the network, the repair strategy of the self-healing network could follow Figure 19. It shows the flow chart of the repair strategy of a self-healing network based on OTDR and the multi-sensor information fusion algorithm.

Initially, the connectivity of the sensor network is monitored using OTDR technology. If the network is unconnected, it is repaired by using the self-healing nodes; otherwise, the stage of data acquisition is completed. The states of the sensors are then monitored. Some suspect sensors in the network are evaluated by using the multi-sensor information fusion algorithm or other methods. If a number of wrong measurements are incurred because of slipping or the sensing performance deteriorates, the sensors are repaired. Finally, we determine whether the structural safety assessment is running properly. By using multi-sensor information fusion algorithm, we can determine whether the damaged sensors should be repaired. For example, if a sensor is redundant or a sensor that has a large sensing cross-correlation with other sensors fails, then it is no need to repair it.

**Figure 20.**
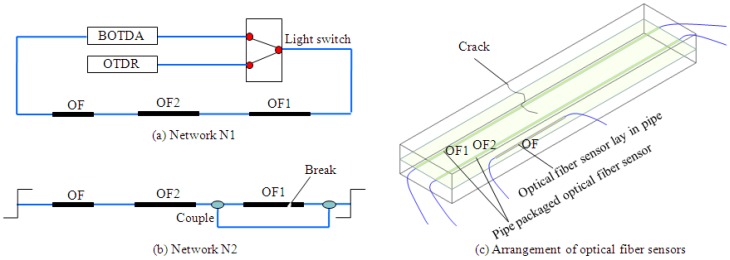
Design and application of primary self-healing network in cement layer. (**a**) Network N1; (**b**) Network N2; (**c**) Arrangement of optical fiber sensors.

**Figure 21.**
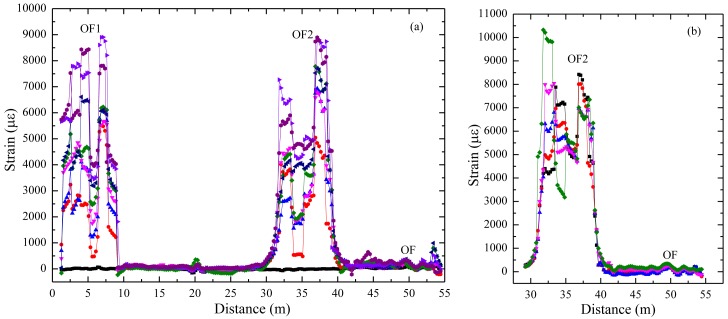
Experimental data: (**a**) Strain measurement of N1; (**b**) Strain measurement of N2.

Based on the demonstration of theory and feasibility provided above, primary networks have been designed to investigate the validation of application in pavement. When one break happens, spare patch cord assisted by couples, is put into use to keep the network available. The original network as shown in Figure 20a is denoted as N1, and the repaired network expressed in Figure 20b is N2. One cement stabilized gravel layer embedded with distributed OF sensors, OF number OF1 and OF2, has been established, with the layout displayed in Figure 20c. Prefabricated cracks have been located on one side of the layer. Light couplers or light switchers have been utilized as the robust link node or emergency component. OF1 sensor is likely to fail initially, when cracks extend from one side to another, as displayed in Figure 20c. The data collected in the experiment is shown in Figure 21. When the strain sensed by OF1 reaches 9,000 με, a break occurs and the repaired network N2 is been enabled to continue the detection. Clearly, the sensing network has always maintained good connectivity. The data indicates that the strain data has good consistency and detection in a vulnerable region is feasible, which means it is reliable to employ a self-healing network for full-scale monitoring of pavements.

## 4. Full-Scale Demonstration Tests on Site

**Figure 22.**
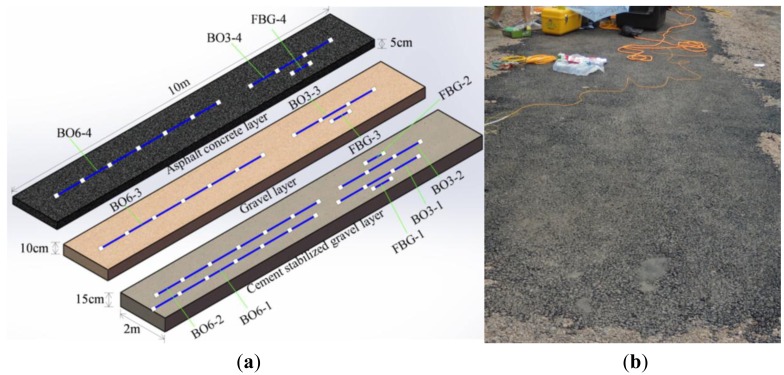
The layout and external view of armoring-wire encapsulated strain sensors in three-layered pavement. (**a**) The layout of strain sensors; (**b**) External view of the three-layered pavement.

Varied types of sensors have been displayed in the sections stated above, and the corresponding performance inspected in laboratory tests has been validated to satisfy the functionality required. Given this, armoring-wire encapsulated strain sensors, including both OF and FBG sensors have been installed in a three-layered asphalt pavement on site, as shown in Figure 22. An OF sensor with six sensing parts is denoted by BO6, and an OF sensor with three sensing parts is labeled as BO3. Due to the impudent paving installation and immature embedding technology, 30 percent of the quasi-distributed OF sensors are not working. Due to the maintenance of BOTDA during the experiments, most of the distributed strain data has not been collect. Therefore, most of the complete data measured just comes from the FBG sensors. No vehicle load is imposed on the structure, and the influence of temperature variation on the strain of the asphalt pavement has been highlighted.

**Figure 23.**
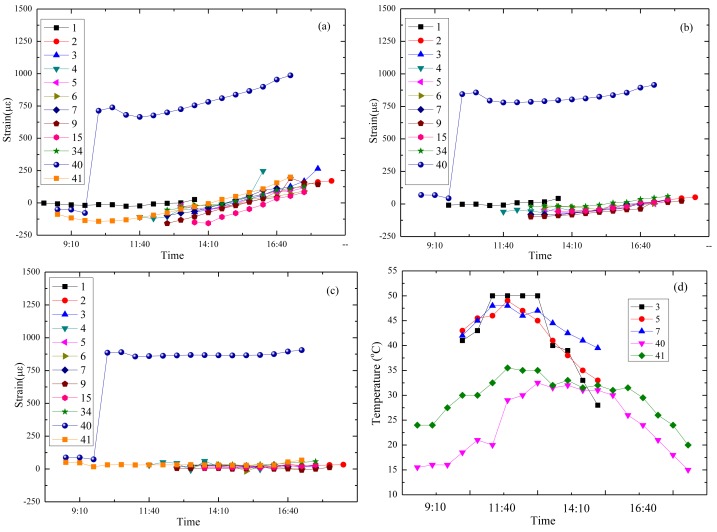
Strain detected by FBG sensors: (**a**) microstrain of the cement stabilized gravel layer by FBG-2; (**b**) microstrain of the gravel layer by FBG-3; (**c**) microstrain of the asphalt concrete layer by FBG-4; (**d**) ground temperature distribution on select days.

Data detected by FBG sensors located at different layers over several days is displayed in Figure 23a–c. The vertical axis represents microstrain, με, and the abscissa axis stands for the corresponding time on one day from morning to afternoon. Ground temperature measured by a thermometer is listed in Figure 23d. Numbers in Figure 23 present the days since the sensors were embedded in the multi-layer pavement structure. For example, number 34 means strain measured by the sensors on the 34th day.

Based on the continuous strain information of the asphalt pavement under environmental effects, it can be seen that temperature played a leading role, and some outcomes could be achieved: strain of the base course in Figure 23a has a small growth in the afternoon, as most of the data is collected in late summer and the temperature of the cement stabilized gravel layer increases after exposing it to sunlight for 10 h; in the strains of the middle layer and surface layer displayed in Figure 23b,c the changes are much smaller, as the heat in the asphalt coarse and gravel layer is much easier to spread; strain at the 40th day in the three layers has a mutation compared with that of other days, and the corresponding temperature is the lowest, which obviously expresses that the strain of asphalt pavement is highly affected by temperature variations and low temperatures are liable to cause large strains (as the weather the day before was hot and a sudden decrease happened on 40th day, the large strain occurred; however, the case on the 41st day was different. The temperature mutation was not so sharp on the 41st day compared with the day before, which explained the low strain phenomenon); the surface layer is most likely to be damaged first under mutation caused by environmental action for the limited tension force that the coarse asphalt could bear.

## 5. Discussion and Conclusions

Due to the complex characteristics of pavement structures, few available references provide definite specifications on their mechanical properties, and SHM is regarded as a feasible way to abstract this information. Optical fiber been selected as sensing element, but its design should take the special features of asphalt pavement into consideration. A network system is required to obtain the full-scale information about pavement structures. Four kinds of raw material and armoring-wire packaged sensors containing both FBG and OF sensors have been put forward and lab tests have been carried out to study the feasibility of the proposed sensors. These sensors packaged with flexible materials show high “survival rate” after installation. The field test data also proves the potential of using OF-based sensors for pavement structure shrinkage and life cycle performance monitoring over a wide range. Furthermore, the basic theory of a self-healing OF sensing network system is presented, and tests on one cement stabilized gravel layer embedded with OF sensors have been carried out, the results of which have validated the feasibility of its application in pavement structures.

In one word, the research in this article provides a valuable method and meaningful guidance for the integrity monitoring of civil structures, especially pavements. Since the work presented in this paper focuses on exhibiting the preliminary achievement of each topic, much deeper research and improvements will be performed in the future. For example, the accuracy of armoring-wire packaged quasi-distributed OF sensors and the strain transfer error between sensor core and pavement (host material) will be analyzed and explained. However, the current results already show the feasibility and prospects of the proposed sensors and self-healing network sensing system.

## Figures and Tables

**Table 1.** Gradation of the fine aggregate asphalt mixture.

**Size of Sieve Pore (mm)**		**<0.075**		**0.075**		**0.15**		**0.3**		
Passing Percent for Sieve Pore (%)		0		24		33		65		
Percent of Gradation (%)		24		9		32		35	

**Table 2.** The aggregates of asphalt mastic.

**Titles**		**Components**						
Sieve diameter (mm)		0.075		0.15		0.3		
Wight of mineral aggregate (g)		90		320		350	

## References

[1] Housner G.W., Bergman L.A., Caughey T.K., Chassiakos A.G., Claus R.O., Masri S.F., Skelton R.E., Soong T.T., Spencer B.F., Yao J.T.P. (1997). Structural control past, present and future. J. Eng. Mech..

[2] Mufti A. (2006). Guidelines for Structural Health Monitoring.

[3] Ansari F. (2005). Sensing Issues in Civil Structural Health Monitoring.

[4] Farrar R.C., Worden K. (2007). An introduction to structural health monitoring. Philos. Trans. R. Soc. A Math. Phys. Eng. Sci..

[5] Brownjohn J.M.W. (2007). Structural health monitoring of civil infrastructure. Philos. Trans. R. Soc. A Math. Phys. Eng. Sci..

[6] Ou J.P. (2007). Research and practice of intelligent sensing technologies in civil structural health monitoring in the mainland of China. Int. Soc. Opt. Eng..

[7] Majumder M., Gangopadhyay K.T., Chakraborty K.A., Dasgupta K., Bhattacharya K.D. (2008). Fiber Bragg gratings in structure health monitoring-present status and applications. Sens. Actuator A Phys..

[8] Li H.N., Gao D.W., Yi T.H. (2008). Advances in structural health monitoring systems in civil engineering. Adv. Mech..

[9] Annmadas K.K.K., Annamdas V.G.M. (2010). Review on developments in fiber optical sensors and applications. SPIE Def. Secur. Sens..

[10] Li H., Ou J.P. (2011). Structural health monitoring: From sensing technology stepping to health diagnosis. Procedia Eng..

[11] Lin W.T., Zhang C.X., Li L.J., Liang S. (2012). Review on development and applications of fiber optic sensors. Photonics Optoelectron. (SOPO) Symp..

[12] Yasin M., Harun W.S., Arof H. (2012). Fiber Optic Sensors.

[13] Alwis L., Sun T., Grattan V.T.K. (2013). Optical fibre-based sensor technology for humidity and moisture measurement: Review of recent recent progress. Measurement.

[14] Sabri N., Aljunid S.A., Salim M.S., Ahmad R.B., Kamaruddin R. (2013). Toward optical sensors: Review and applications. J. Phys. Conf. Ser..

[15] Cusano A., Cutolo A., Albert J. (2011). Fiber Bragg Grating Sensors: Research Advancements, Industrial Applications and Market Exploitation.

[16] Chen J.J., Liu B., Zhang H. (2011). Review of fiber Bragg grating sensor technology. Front. Optoelectron. China.

[17] Xu G.Q., Xiong D.Y. (2013). Applications of fiber Bragg grating sensing technology in engineering. Chin. Opt..

[18] Ecke W., Schmitt W.M. Fiber Bragg gratings in industrial sensing.

[19] Mason B., Hogg D., Measures R.M. Fiber optic strain sensing for smart adaptive structures.

[20] Kundu P., Ramakrishna C., Saxena V.N. (2013). Optical fiber sensors for smart structures: A review. Def. Sci. J..

[21] Frangopol M.D. (2011). Life-cycle performance, management, and optimization of structural systems under uncertainty: Accomplishments and challenges. Struct. Infrastruct. Eng..

[22] Zhi Z., Ou J.P. (2005). Development of FBG Sensors for Structural Health Monitoring in Civil Infrastructures. Sens. Issues Civ. Struct. Health Monit..

[23] Ansari F. (2007). Practical implementation of optical fiber sensors in civil structural health monitoring. J. Intell. Mater. Syst. Struct..

[24] Ou J.P., Zhi Z. (2007). Optic fiber Bragg grating based sensing technologies and their applications in structural health monitoring. SPIE Int. Soc. Opt. Eng..

[25] Dorman G.M. (1965). Design curves for flexible pavements based on layered system theory. Highw. Res. Rec..

[26] Cheng H.D., Miyojim M. (1998). Novel system for automatic pavement distress detection. J. Comput. Civ. Eng..

[27] Rajbongshi P., Das A. (2009). Estimation of temperature stress and low-temperature crack spacing in asphalt pavments. J. Trans. Eng..

[28] Singh A., Das A., Basu S. (2012). A numerical study on the effect of aggregate gradation on mechanical response of asphalt mix. KSCE J. Civ. Eng..

[29] Sha A.M., Tu S. (2012). Cracks characteristics and damage mechanism of asphalt pavement with semi-rigid base. 7th RILEM International Conference on Cracking in Pavements.

[30] Surve R.S. (2003). Interferometric Optical Fiber Sensor for Highway Pavements and Civil Structures. Ph.D. Thesis.

[31] Dai Q.L., Sadd H.M., Parameswaran V., Shukla A. (2005). Prediction of damage behaviors in asphalt materials using a micromechanical finite-elment model and image analysis. J. Eng. Mech..

[32] Goktepe B.A., Agar E., Lav H.A. (2006). Advances in back calculating the mechanical properties of flexible pavements. Adv. Eng. Softw..

[33] Zhang Q.S. (1985). Experimental verification of the elastic layer system theory and its application. China Civ. Eng. J..

[34] Hudson W.R., Uddin W. Future pavement evaluation technologies: Prospects and opportunities.

[35] Gao J.Q., Shi B., Zhang W., Ke M.Y., Liu H.X., Zhang D. (2005). Application of distributed fiber optic sensor to bridge and pavement health monitoring. J. Disaster Prev. Mitig. Eng..

[36] Zhou Z., Liu W.Q., Huang Y., Wang H.P., He J.P., Huang M.H., Ou J.P. (2012). Optical fiber Bragg grating sensor assembly for 3D strain monitoring and its case study in highway pavement. Mech. Syst. Signal Process..

[37] Chen S.X., Zhang X.N., Xu Q.L., Meng S.T. (2006). Experiment and research of grating strain sensor on asphalt pavement. Chin. J. Sens. Actuator.

[38] Malla B.R., Sen A., Garrick W.N. (2008). A special fiber optic sensor for measuring wheel loads of vehicles on highways. Sensors.

[39] Qian Z.D., Huang W., Guan Y.S., Han G.Y. (2008). Application of BOTDA on cracking monitoring for asphalt concrete pavement. J. Southeast Univ..

[40] Tan Y.Q., Dong Z.J., Tian G.L., Hu Q.L. (2009). Evaluating method of the coordination deformation between asphalt mixture and fiber Bragg grating sensor. J. Civ. Archit. Environ. Eng..

[41] Wang C., Hu Q.L., Lu Q.Y. (2012). Research on a novel low modulus OFBG strain sensor for pavement monitoring. Sensors.

[42] Wang J.N., Tang J.L. (2010). Feasibility of fiber Bragg grating and long-period fiber grating sensors under different environmental conditions. Sensors.

[43] Xie J.G., Wang Q.T., Liu S.L., Li K., Zhang S. (2011). Experimental study on strain monitoring of asphalt concrete using FBG (Fiber Bragg Grating). J. Chang'an Univ..

[44] Artieres O., Bacchi M., Bianchini P., Hornych P., Dortland G. (2012). Strain measurement in pavements with a fibre optics sensor enabled geotextile. 7th RILEM International Conference on Cracking in Pavements.

[45] Whelan B.E., Brunton M., Nosenzo G., Kay D., Buys H. Continuous monitoring of mining induced strain in a road pavement using fibre bragg grating sensors.

[46] Grellet D., Dore G., Bilodeau J.-P. (2012). Comparative study on the impact of wide base tires and dual tires on the strains occuring within flexible pavements asphalt concrete surface course. Can. J. Civ. Eng..

[47] Loizos A., Plati C., Papavasilio V. (2013). Fiber optic sensors for assessing strains in cold in-place recycled pavements. Int. J. Pavement Eng..

[48] Xue W.J., Wang D., Wang L.B. (2012). A review and perspective about pavement monitoring. Int. J. Pavement Res. Technol..

[49] Zhou Z., He J.P., Xiao H., Ou J.P. (2013). A Novel Self-healing Optical Fiber Network. Appl. Mech. Mater..

[50] (2000). Standard Test Methods of Bitumen and Bituminous Mixtures for Highway Engineering.

[51] (2005). Test Methods of Aggregate for Highway Engineering.

[52] Lan C.G. (2012). Life-Cycle Monitoring of Pre-Stress Loss and Safety Assessment for RC Structure. Ph.D. Thesis.

[53] Zhang H., Wu Z.S., Kentaro I. (2007). Performance evaluation of BOTDA based distributed optic fiber sensors for crack monitoring. Strain.

[54] Bao X.Y., Chen L. (2011). Recent progress in brillouin based fiber sensors. Sensors.

[55] Yuan H.Q., Yuan J., Du J. (2003). The sensing principle of FBG and its experimental application in structure strengthening detection. J. Wuhan Univ. Technol. Mater. Sci..

[56] Zhang A.P., Gao S.R., Yan G.Y., Bai Y.B. (2012). Advances in optical fiber Bragg grating sensor technologies. Photonic Sens..

[57] Wang H.P., Liu W.Q., Zhou Z., Wang S.H., Li Y., Guo Z.W. The behavior of a novel raw material-encapsulated FBG sensor for pavement monitoring.

[58] Wang H.P., Zhou Z. (2012). A Review on Strain Transfer Mechanism of Optical Fiber Sensors. Pac. Sci. Rev..

[59] LeBlanc M. (1999). Interaction Mechanics of Embedded Single-Ended Optical Fibre Sensors Using Novel *In-Situ* Measurement Techniques. Ph.D. Thesis.

[60] Wang H.P., Zhou Z., Liu W.Q., Li X. Optimization analysis and experimental validation of distributed optical fiber sensors for pavement monitoring based on strain transfer mechanism.

[61] Zhou Z., He J.P., Ou J.P. (2012). A novel self-healing optical fiber network. Pac. Sci. Rev..

